# Homocysteine Causes Endothelial Dysfunction via Inflammatory Factor-Mediated Activation of Epithelial Sodium Channel (ENaC)

**DOI:** 10.3389/fcell.2021.672335

**Published:** 2021-06-17

**Authors:** Chen Liang, Qiu-Shi Wang, Xu Yang, Di Zhu, Yu Sun, Na Niu, Jie Yao, Bi-Han Dong, Shuai Jiang, Liang-Liang Tang, Jie Lou, Chang-Jiang Yu, Qun Shao, Ming-Ming Wu, Zhi-Ren Zhang

**Affiliations:** Departments of Pharmacy and Cardiology, Harbin Medical University Cancer Hospital, Institute of Metabolic Disease, Heilongjiang Academy of Medical Science, Heilongjiang Key Laboratory for Metabolic Disorder and Cancer Related Cardiovascular Diseases, NHC Key Laboratory of Cell Transplantation, Harbin Medical University and Key Laboratories of Education Ministry for Myocardial Ischemia Mechanism and Treatment, Harbin, China

**Keywords:** hyperhomocysteinemia, endothelial epithelial sodium channel, inflammation and cyclooxygenase-2, reactive oxygen species, vascular dysfunction

## Abstract

**Background:**

Hyperhomocysteinemia (HHcy) causes cardiovascular diseases via regulating inflammatory responses. We investigated whether and how the epithelial sodium channel (ENaC), a recently identified ion channel in endothelial cells, plays a role in HHcy-induced endothelial dysfunction.

**Methods:**

Cell-attached patch-clamp recording in acute split-open aortic endothelial cells, western blot, confocal imaging, and wire myograph combined with pharmacological approaches were used to determine whether HHcy-mediated inflammatory signaling leads to endothelial dysfunction via stimulating ENaC.

**Results:**

The data showed that 4 weeks after L-methionine diet the levels of plasma Hcy were significantly increased and the ENaC was dramatically activated in mouse aortic endothelial cells. Administration of benzamil, a specific ENaC blocker, ameliorated L-methionine diet-induced impairment of endothelium-dependent relaxation (EDR) and reversed Hcy-induced increase in ENaC activity. Pharmacological inhibition of NADPH oxidase, reactive oxygen species (ROS), cyclooxygenase-2 (COX-2)/thromboxane B2 (TXB2), or serum/glucocorticoid regulated kinase 1 (SGK1) effectively attenuated both the Hcy-induced activation of endothelial ENaC and impairment of EDR. Our *in vitro* data showed that both NADPH oxidase inhibitor and an ROS scavenger reversed Hcy-induced increase in COX-2 expression in human umbilical vein endothelial cells (HUVECs). Moreover, Hcy-induced increase in expression levels of SGK-1, phosphorylated-SGK-1, and phosphorylated neural precursor cell-expressed developmentally downregulated protein 4-2 (p-Nedd4-2) in HUVECs were significantly blunted by a COX-2 inhibitor.

**Conclusion:**

We show that Hcy activates endothelial ENaC and subsequently impairs EDR of mouse aorta, via ROS/COX-2-dependent activation of SGK-1/Nedd4-2 signaling. Our study provides a rational that blockade of the endothelial ENaC could be potential method to prevent and/or to treat Hcy-induced cardiovascular disease.

## Introduction

As an independent risk factor, hyperhomocysteinemia (HHcy) is closely associated with coronary heart disease, venous and arterial thrombosis, atherosclerosis, and hypertension (Rodrigo et al., 2003; Ganguly and Alam, 2015). However, the underlying molecular mechanisms of HHcy-induced endothelial dysfunction remain to be elucidated.

Previous studies showed that homocysteine (Hcy) led to vascular dysfunction by regulating a variety of ion channels, including big- (BK_*Ca*_), intermediate- (IK_*Ca*_), and small-conductance (SK_*Ca*_) Ca^2+^-activated K^+^ channels. For instance, Hcy inhibited BK_*Ca*_ channels in a dose-dependent manner in human umbilical vein endothelial cells (Zhang et al., 2005). Moreover, Hcy induced porcine coronary endothelial dysfunction through ER stress-mediated inhibition of SK_*Ca*_ and IK_*Ca*_ channels (Wang et al., 2015). In addition, studies showed that Hcy significantly inhibited BK_*Ca*_ channels in isolated human and rat artery smooth muscle cells and that the effects of Hcy on BK_*Ca*_ channels were reversed by the DPI, an inhibitor of NADPH oxidase (Cai et al., 2007). Furthermore, Hcy also suppressed BK_*Ca*_ channel probably by activating NADPH oxidase in porcine coronary smooth muscle cells. However, whether Hcy regulates endothelial epithelial sodium channel (ENaC), a newly identified ion channel in endothelium, remains unclear.

Previous studies showed that the activation of endothelial ENaC causes vascular stiffness (Guo et al., 2016; Tarjus et al., 2017), which is the first indication of the regulatory role of ENaC in vascular function. The later functional studies showed that endothelial ENaC plays an important role in a variety of pathological stimuli including oxidized LDL-, high fat diet-, and high salt diet-induced endothelial dysfunction and impairment of endothelium-dependent relaxation (EDR) (Liang et al., 2018; Wang et al., 2018b; Yang et al., 2020; Niu et al., 2021). We recently showed that manipulating the expression levels of endothelial ENaC or pharmacological blockade of ENaC alleviates high fat diet-induced production and secretion of proinflammatory cytokines, including TNF-α, IL-1β, IL-6, thereby reduces the formation of atherosclerotic lesion in LDL receptor knockout (LDLr^–/–^) mice (Niu et al., 2021). These results strongly suggest that activation of endothelial ENaC is tightly linked to inflammatory factor-mediated dysfunction of endothelial dysfunction and pathogenesis of atherosclerosis. These results led us to hypothesize that endothelial ENaC may involve in Hcy-induced vascular dysfunction via ROS accumulation and activation of inflammatory signaling.

It has been well documented that cyclooxygenase-2 (COX-2), a pro-inflammatory factor, is involved in inflammation-mediated endothelial dysfunction (Tian et al., 2012; Zhang et al., 2018). COX-2 converts arachidonic acid into prostanoids, including thromboxane, prostaglandin, and prostacyclins. The productions of pro-inflammatory prostanoids such as thromboxane A2 (TXA2) and prostaglandin E2 (PGE2) are the main pathways, where COX-2 elicits the onset of inflammation. Recent studies indicate that COX-2 is highly expressed in endothelial cells in response to different stimuli such as angiotensin II (Niazi et al., 2017), palmitate (Gao et al., 2014), and bone morphogenic protein 4 (Wong et al., 2010) in hypertensive and diabetic models. These results suggest that COX-2 plays a role in these stimuli-induced endothelial dysfunctions. In addition, it has been reported that Hcy-induced increase in COX-2 expression contributes to inflammatory processes in murine macrophages (Lee et al., 2013), chondrocytes (Ma et al., 2018), and hepatic cells (Wu et al., 2009). More importantly, studies showed that celecoxib, a selective COX-2 inhibitor, improves endothelial function and reduces the systemic inflammatory response in coronary artery disease (Chenevard et al., 2003). Consistently, our recent study provides a clue that high fat diet-induced activation of ENaC may regulate COX-2-dependent inflammatory signaling (Niu et al., 2021).

In this study, we investigated the role of endothelial ENaC and underlying mechanisms in HHcy-induced vascular dysfunction, using L-methionine administration-induced HHcy mouse model combined with a variety of experimental approaches. We show that Hcy leads to vascular dysfunction by stimulating ENaC via ROS/COX-2-mediated activation of SGK1/Nedd4-2 signaling.

## Materials and Methods

### Animals

All animal care and experimental procedures were approved by the Animal Research Ethical Committee of Harbin Medical University. All studies involving animals are reported conformed to the ARRIVE guidelines for reporting experiments (Kilkenny et al., 2010; McGrath et al., 2010).

C57BL/6J mice were purchased from the animal center of the second affiliated hospital of Harbin Medical University (Harbin, China). Male C57BL/6J mice aged 8–10 weeks (18–20 g) were fed with standard laboratory chow and had free access to water under a 12-h light/dark cycle with the ambient humidity at 50–80% and the controlled temperature at 22–24°C. C57BL/6J mice were given 2% (wt/wt) L-methionine in a chow diet for 4 weeks to establish the HHcy mouse model and the plasma levels of Hcy were significantly elevated. We also treated the aortic arteries and HUVECs with 100 μM Hcy to mimic the HHcy in mice, as previously described (Zhang et al., 2012). Mice were intragastrical administrated with benzamil (1 mg/kg/day) for 4 weeks, as previously described (Niu et al., 2021). C57BL/6J mice were randomly divided into four groups as follows: control, standard chow; HHcy, 2% (wt/wt) L-methionine diet; HHcy plus benzamil; and benzamil alone. Four weeks later, all animals were anesthetized, blood was collected, and the aorta were isolated for performing the experiments described down below. Plasma concentrations of Hcy and TXB2 were measured by the ELISA kits (Nan Jing Jian Cheng Biotech Co., Ltd., China) according to the manufacturer’s guidelines. The measurements were performed in multiple duplications. The produced color intensity was assessed at a wavelength of 450 nm using a multifunctional microplate reader (SpectraMax M5, Sunnyvale, MD, United States).

### Cell Culture

Human umbilical vein endothelial cells were cultured in endothelial cell growth medium (Hyclone, United States) supplemented with 10% FBS (Hyclone, United States) and 1% penicillin/streptomycin (Invitrogen, United States), as described previously (Zheng et al., 2016). When HUVECs had grown to 80–90% confluency in six-well plates, they were incubated with indicated reagents and maintained at 37°C under 95% air and 5% CO_2_. Cells were used within 7–9 passages.

### COX-2 Gene Silencing

One day prior to infection, HUVECs were cultured in the six-well plates until the density of the cells was achieved to 20–30%. The HUVECs were, respectively, transfected with either COX-2-shRNA green fluorescent protein (GFP) lentivirus (COX-2 gene silencing group) or with a scrambled shRNA GFP lentivirus (control group), in the presence of HiTransG A in order to enhance the virus infection. Three days post transfection, the gene silencing efficiency was examined by quantitative real-time polymerase chain reaction (qRT-PCR) and Western blot assays. The sequences of COX-2-shRNA and control were as follows: 5′-GCAGATGAAATACCAGTCTTT-3′ and, 5′- TTCTCCGAACGTGTCACGT-3′. The lentiviruses were purchased from Shanghai GeneChem Co., Ltd. (Shanghai, China). The infected HUVECs were, respectively, treated with 100 μM Hcy for 6 h, followed by testing the expression levels of t-SGK1, p-SGK1, t-Nedd4-2, and p-Nedd4-2.

### *In situ* Patch-Clamp Recording

As described previously (Liu et al., 2015; Liang et al., 2018), *in situ* patch-clamp recordings of ENaC single-channel currents were performed in intact vascular endothelia. Each dissected aortic pectoralis was placed in a Petri dish containing physiological salt solution (PSS). They were then placed on a 5 × 5 mm cover glass coated with L-polylysine and transferred into a recording chamber mounted on an inverted Nikon microscope (Tokyo, Japan), allowing direct access to the endothelial cell layer.

The single-channel ENaC currents were recorded in a cell-attached configuration with an Axon Multiclamp 200B amplifier (Axon Instruments; United States) connected to a PC running Clampex 10.2 software at room temperature (22–24°C). Patch pipettes were fabricated from borosilicate glass capillaries using a Sutter P-97 horizontal puller. The pipettes had the resistance of ranged from 6 to 10 MΩ when filled with the pipette solution containing (in mM) 135 NaCl, 4.5 KCl, 0.1 EGTA, 5 HEPES, and 5 Na-HEPES (pH 7.2; adjusted with NaOH). The bath solution, contained (in mM) 135 NaCl, 4.5 KCl, 1 MgCl_2_, 1 CaCl_2_, 5 HEPES, and 5 Na-HEPES (pH 7.2; adjusted with NaOH). Single-channel currents were recorded immediately after gigaseal formation for at least 15 min. The data were recorded by application of 0 mV to the patch pipettes at 5 kHz with a low-pass filter at 1 kHz. Prior to analyses, single-channel traces were further filtered at 30 Hz and single-channel events were listed and values of the ENaC open probability (*P*_*O*_) were analyzed by using Clampfit 10.2 software.

### qRT-PCR and Western Blot Analysis

For western blot analyses were performed in HUVECs in the absence or in the presence of 100 μM Hcy for 6 h. Cell lysates were centrifuged at 12,000 rpm at 4°C for 15 min to remove debris. Protein concentration was assayed by BCA Protein Assay Kit (APPLYGEN, China). Protein samples were electrophoresed through 10% SDS-polyacrylamide gels and then transferred to nitrocellulose membranes using a *Trans-*blot unit (Bio-Rad Laboratories) for 90 min at 300 mA. Membranes were incubated in 5% (wt/vol) non-fat milk dissolved in 1 × TBS-T for 1 h at room temperature (22–24°C) to block non-specific binding sites. Then, the membrane was probed with the primary antibodies against COX-1 (1:1,000, ab109025, Abcam, United Kingdom), COX-2 (1:1,000, ab52237, Abcam, United Kingdom), SGK1 (1:500, ab59337, Abcam, United Kingdom), p-SGK1 (1:500, ab55281, Abcam, United Kingdom), Nedd4-2 (1:10,000, ab131167, Abcam, United Kingdom), p-Nedd4-2 (1:1,000, ab168349, Abcam, United Kingdom), or GAPDH (1:10,000, ab8245, Abcam, United Kingdom) overnight at 4°C. The membranes were washed in TBS-T and subsequently incubated with goat anti-rabbit IRDye^®^ 800 CW (1:10,000, P/N 926-32211, LI-COR) or goat anti-mouse IRDye^®^ 800 CW (1:10,000, P/N 926-32210, LI-COR) at room temperature (22–24°C) for another 1 h. Membranes were finally washed again in TBS-T and the protein bands were detected by the Odyssey infrared imaging system (LI-COR) and Odyssey v3.0 software.

Total RNA was extracted from HUVECs using TRIzol reagent (Invitrogen, Carlsbad, CA, United States). Reverse transcription was performed with the RT system protocol in a 20 μL reaction mixture, similar to that described previously (Wu et al., 2014; Niu et al., 2021). Total RNA (1 μg) was used in the reaction, and a random primer was used for the initiation of cDNA synthesis. The reaction mixture was incubated at 25°C for 10 min, 37°C for 120 min and 85°C for 5 min. RT-PCR was performed by an ABI Prism 7500 sequence detection system using SYBR Green PCR core reagents (Bimake). PCR was performed by following the manufacturer’s recommendations for a 25 μL reaction volume. Transcript quantities were compared by using the relative quantitation method, where the amount of detected mRNA was normalized to the amount of endogenous control (GAPDH) mRNA. The value relative to the control sample value is given by 2^–ΔΔ*CT*^. Expression levels of mRNA were determined using the following primers: COX-2-human-Forward (GCTCAGCCATACAGCAAATC), COX-2-human-Reverse (TGTGTTTGGAGTGGGTTTCA), GAPDH-human-Forward (CAACTTTGGTATCGTGAAGG), GAPDH- human -Reverse (AGAGGCAGGGATGATGTTCTG).

### Isometric Force Measurement in a Wire Myograph

Myograph function analysis was performed as previously described (Wong et al., 2010; Liang et al., 2018; Niu et al., 2021). Male C57BL/6J mouse aortic pectoralis was gently isolated and then placed in oxygenated ice-cold Krebs solution (composition in mM: 119 NaCl, 4.7 KCl, 2.5 CaCl_2_, 1 MgCl_2_, 25 NaHCO_3_, 1.2 KH_2_PO_4_, and 11 D-glucose, pH 7.2–7.4). Each aorta was stripped of surrounding connective tissues under a dissecting microscope and cut into 2 mm length ring segments. Rings were exposed to 100 μM Hcy with or without each of the following inhibitor for 6 h: benzamil (0.5 μM, ENaC blocker), TEMPOL (100 μM; ROS scavenger), apocynin (100 μM, NADPH oxidase inhibitor), sc560 (0.3 μM; COX-1 inhibitor), celecoxib (3 μM; COX-2 inhibitor), furegrelate (10 μM; thromboxane synthase inhibitor), and GSK650394 (10 μM; SGK1 inhibitor). The aortic pectoralis was mounted to a wire myograph system (Danish Myo Technology, Aarhus, Denmark) and bathed in oxygenated Krebs solution at 37°C. Each ring was stretched to 3 mN and then allowed to equilibrate for 1 h before the myograph experiment. After the stabilization period, KPSS (containing 60 mM K^+^) was added to the chambers and washed out with Krebs solution until a reproducible maximal contraction was achieved. Endothelium-dependent relaxations were studied in phenylephrine (1 μM) pre-contracted endothelium-intact segments in response to cumulative addition of acetylcholine (ACh, in a range of concentration from 1 nM to 10 μM). Sodium nitroprusside (SNP, in a range of concentration from 1 nM to 10 μM), an exogenous NO donor, was used to examine endothelium-independent relaxation.

### Confocal Laser Scanning Microscopy Analysis

Confocal microscopy (Olympus Fluoview 1200, Japan) studies were performed as previously described (Tian et al., 2012; Wang et al., 2018a). Aortic segments were fresh-frozen in optimal cutting temperature compound and sectioned at 5 μm with a freezing microtome (CryoStar NX70, Thermo Fisher Scientific, Waltham, MA, United States). Cryosections were incubated in dihydroethidium (DHE) (Ex515/Em585 nm, 5 μM; Invitrogen, United States) in dark at 37 °C for 15 min. All slides were washed with PBS twice and imaged using a confocal microscope. The signal density was analyzed by ImageJ software (Java-based imaging processing program, National Institute of Health, United States).

5-(and-6)-carboxy-2′,7′-dichlorodihydrofluorescein diacetate (carboxy-H2DCFDA, Invitrogen, United States) was used as the membrane-permeable ROS-sensitive fluorescent indicator, that becomes fluorescent when oxidized. HUVECs grown on confocal dishes were loaded with 2.5 μM carboxy-H2DCFDA for 1 h. Before application of indicated reagents, HUVECs were treated with an iron chelator, 50 μM 2,2′-dipyridyl which suppresses the damaging Fenton reaction for 3 min (Shatalin et al., 2011). Next, labeled cells were washed twice in modified PBS before confocal microscopy analysis. Excitation at 488 nm and emission at 520 nm were used to evaluate the amount of intracellular ROS level in response to indicated reagents.

### Chemicals and Reagents

Unless otherwise noted, all chemicals and reagents were purchased from Sigma-Aldrich. GSK650394 was purchased from Tocris and furegrelate was purchased from Cayman. Acetylcholine, phenylephrine, and TEMPOL were prepared in distilled water and the others in DMSO. TEMPOL and SNP were prevented from light during preparation and experiments.

### Data Analysis

All data are presented as the mean values ± SEM. Statistical analysis was performed with GraphPad Prism 5 software (GraphPad; La Jolla, CA) for all statistical calculations. The student’s *t*-test was used between two groups. Analysis of variance was used for multiple comparisons. In the cases, where ANOVA was used, a *post hoc* test (Bonferroni) was used. The results were considered significant at *P* < 0.05.

## Results

### Blockade of ENaC by Benzamil Reverses Vascular Dysfunction in L-Methionine-Treated Mice

**FIGURE 1:**
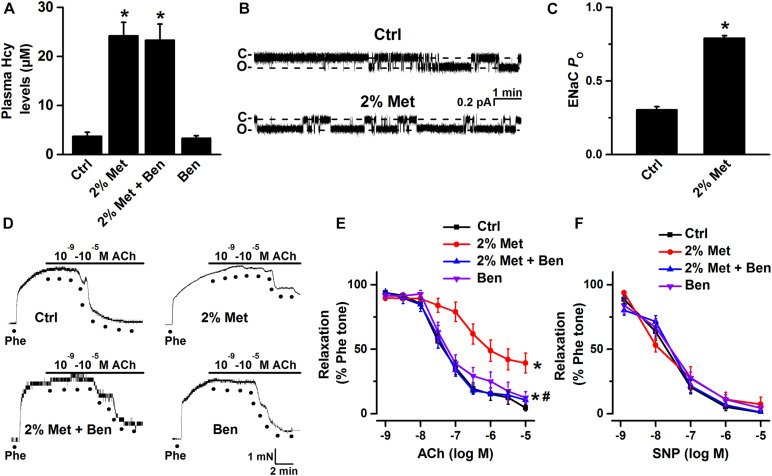
L-methionine-induced HHcy activated aortic endothelial ENaC and impaired EDR was reversed by blockade of ENaC. **(A)** Mean plasma Hcy levels were measured in control and L-methionine (Met)-treated mice with or without benzamil (Ben) treatment. Data are means ± SEM of six mice. *P < 0.05 vs. ctrl. **(B)** Representative ENaC single-channel currents were recorded in endothelial cells of the split-open aorta isolated from control (Ctrl) and L-methionine-treated mice. **(C)** ENaC activity (*P*_*O*_) summarized from the experiments as shown in **(B)**. Data are means ± SEM of six mice. *P < 0.05 vs. ctrl. **(D)** Representative traces obtained from wire myograph assays and **(E)** summarized data of acetylcholine (ACh)-induced relaxation of aorta pectoralis from control and L-methionine-treated mice with or without benzamil treatment. The first dot indicates the application of 10^–9^ M ACh to the 1 μM phenylephrine (Phe) precontracted arterial rings, whereas the following dots indicate ACh concentrations gradually increased from 10^–8^.^5^ to 10^–5^ M. Data are means ± SEM of six mice. *P < 0.05 vs. ctrl; #*P* < 0.05 vs. 2% Met groups. **(F)** Summary of artery relaxation induced by different doses of sodium nitroprusside (SNP) concentrations ranged from 10^–9^ to 10^–5^ M in control and L-methionine -treated mice with or without benzamil treatment.

We have previously reported that blockade of ENaC has a protective effect on ox-LDL-induced vascular dysfunction in mice (Liang et al., 2018). Here, we investigated whether blockade of endothelial ENaC ameliorated HHcy-induced vascular dysfunction in mice. As shown in Figure 1A, the mean plasma level of Hcy was significantly elevated in L-methionine-treated mice as compared with control; whereas administration of benzamil, a selective ENaC blocker, had no effect on the elevated plasma level of Hcy in L-methionine-treated mice (Figure 1A). Importantly, our patch-clamp data showed that the ENaC activity was significantly increased in intact endothelial cells of the aorta from L-methionine-treated mice, compared with the control (Figures 1B,C). Since our previous data showed that elevation of ENaC activity impaired ACh-induced EDR in the salt-sensitive rats (Wang et al., 2018b), therefore, we hypothesize that blockade of ENaC may prevent Hcy-induced vascular dysfunction in L-methionine-treated mice. As expected, the ACh-induced EDR was dramatically impaired by L-methionine in mice, which was reversed by benzamil; while the ACh-induced EDR was not affected by benzamil (Figures 1D,E). Moreover, neither L-methionine nor benzamil affected SNP-induced endothelium-independent artery relaxation (Figure 1F).

**FIGURE 2:**
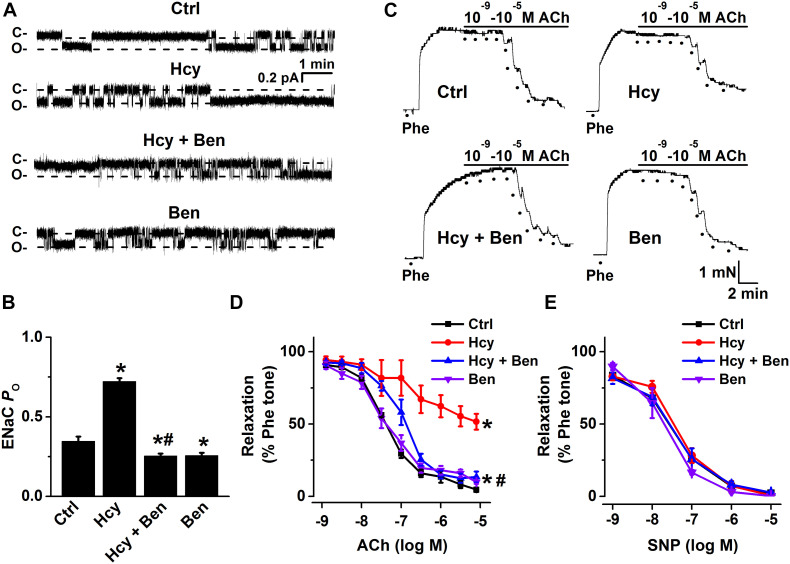
Benzamil attenuates exogenous Hcy-induced increase in endothelial ENaC activity and impairment of EDR in mouse aorta. **(A)** Representative ENaC single-channel currents were recorded from endothelial cells from split-open aorta either under control conditions or, respectively, treated with 100 μM Hcy, 100 μM Hcy plus 0.5 μM benzamil (Ben), or 0.5 μM benzamil alone for 6 h. **(B)** ENaC activity (*P*_*O*_) summarized from the recordings as shown as shown in **(A)**. Data are means ± SEM of six mice. *P < 0.05 vs. ctrl; #*P* < 0.05 vs. Hcy. **(C)** Representative traces obtained from wire myograph assays under each indicated condition (Ctrl, control; Hcy, the isolated aorta treated with Hcy; Hcy + Ben, the aorta incubated with Hcy plus benzamil; Ben, benzamil) and **(D)** summarized data of ACh-induced relaxation of aorta under each condition, as indicated in **(C)**. The first dot indicates the application of 10^–9^ M ACh to the 1 μM Phe precontracted arterial rings, whereas the following dots indicate ACh concentrations in a range of 10^–8^.^5^ to 10^–5^ M. Data are means ± SEM of six mice. *P < 0.05 vs. ctrl; #*P* < 0.05 vs. Hcy. **(E)** Summary of artery relaxation induced by different doses of SNP at the concentrations from 10^–9^ to 10^–5^ M in aorta from the four groups indicated above.

To further confirm that L-methionine-induced activation of ENaC was brought about by HHcy, we treated isolated mouse aorta with 100 μM Hcy for 6 h and performed single-channel recordings intact endothelial cells. Consistent with the data obtained from *in vivo* experiments, our *ex vivo* data demonstrated that exogenous Hcy led to the significantly increased ENaC activity and that the exogenous Hcy-induced increase in ENaC activity was reversed by benzamil (Figures 2A,B). Moreover, exogenous Hcy treatment-induced impairment of EDR was dramatically alleviated by benzamil (benzamil did not affect EDR; Figures 2C,D); whereas exogenous Hcy or benzamil had no effect on SNP-induced endothelium-independent relaxation (Figure 2E). These data together suggest that HHcy stimulates endothelial ENaC and that blockade of endothelial ENaC by benzamil ameliorates HHcy-induced impairment of EDR in mice.

### An NADPH Oxidase Inhibitor or a Superoxide Scavenger Reverses Hcy-Induced Activation of ENaC and Endothelial Dysfunction

**FIGURE 3:**
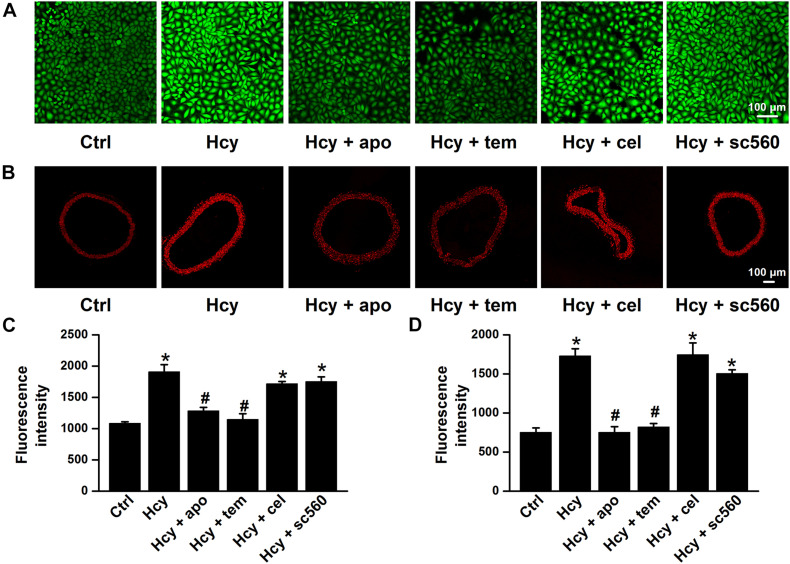
NADPH oxidase inhibitor or TEMPOL reverses exogenous Hcy-induced ROS generation in both HUVECs and mouse aorta. **(A,B)** Representative confocal microscopy images, taken from HUVECs **(A)** or aorta **(B)**, under control condition (Ctrl) or treated with 100 μM Hcy in the absence or in the presence of 100 μM apocynin (apo), 100 μM TEMPOL (tem), 3 μM celecoxib (cel), and 0.3 μM sc560 for 6 h, respectively. **(C,D)** Summarized fluorescent intensities in HUVECs (DCF staining) or aorta (DHE staining), measured from the data shown in **(A,B)**, reflect the ROS levels in HUVECs or aorta. Data are means ± SEM of six individual experiments. *P < 0.05 vs. ctrl; #*P* < 0.05 vs. Hcy.

It has been reported that Hcy induces activation of NADPH oxidase, a major source of ROS produced by endothelial cells, and leads to vascular dysfunction (Babior, 2000; Zeng et al., 2003; Liu et al., 2019), and our previous findings showed that the accumulation of intracellular ROS stimulates ENaC in both aortic endothelial cells and renal epithelial cells (Zhang et al., 2013; Liang et al., 2018; Wang et al., 2018a; Wu et al., 2019). Thus, we examined whether Hcy increased ENaC activity by promoting ROS generation in the intact endothelial cells of the mouse aorta. Indeed, our data showed that exogenous Hcy significantly elevated ROS production in both mouse aorta and HUVECs, as determined by DHE and DCF staining, respectively. In addition, apocynin, an NADPH oxidase inhibitor, and TEMPOL, a superoxide scavenger, significantly inhibited Hcy-induced ROS production in mouse aorta and in HUVECs. Whereas, celecoxib (a COX-2 inhibitor) and sc560 (a COX-1 inhibitor) had no effect on exogenous Hcy-induced oxidative stress (Figures 3A–D).

**FIGURE 4:**
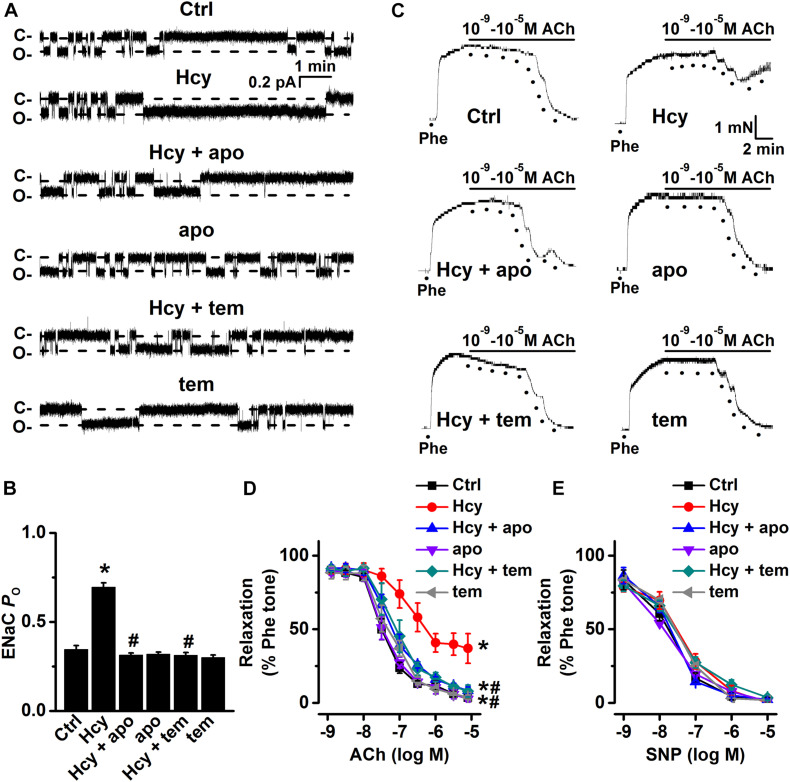
Inhibition of ROS attenuates Hcy-induced increase in ENaC activity and impairment of EDR in mouse aorta. **(A)** Representative ENaC single-channel currents were recorded in endothelial cells from split-open aorta either under control conditions or treated with 100 μM Hcy, 100 μM Hcy plus 100 μM apocynin (apo), 100 μM apo alone, 100 μM Hcy plus 100 μM TEMPOL (tem), or 100 μM TEMPOL alone for 6 h, respectively. **(B)** Summarized ENaC *P*_*O*_ obtained from recordings as shown in **(A)**. Data are means ± SEM of six mice. *P < 0.05 vs. ctrl; #*P* < 0.05 vs. Hcy. **(C)** Representative traces obtained from wire myograph assays under each indicated condition and **(D)** summarized data of ACh-induced relaxation under each indicated condition. The first dot indicates the application of 10^–9^ M ACh to the 1 μM Phe precontracted arterial rings, whereas the following dots indicate ACh concentrations gradually increasing from 10^–8^.^5^ to 10^–5^ M. Data are means ± SEM of six mice. *P < 0.05 vs. ctrl; #*P* < 0.05 vs. Hcy. **(E)** Summary of artery relaxation induced by different doses of SNP increasing from 10^–9^ to 10^–5^ M in aorta from each indicated group.

Moreover, both apocynin and TEMPOL significantly attenuated exogenous Hcy-induced increases in ENaC activity, without affecting the basal ENaC activity in aortic endothelial cells (Figures 4A,B). Importantly, apocynin or TEMPOL ameliorated exogenous Hcy-induced impairment of aortic EDR (Figures 4C,D), while endothelial-independent relaxations in response to SNP were unaffected by all reagents (Figure 4E). These data suggest that exogenous Hcy increases ENaC activity, which leads to impairment of EDR via promoting the accumulation of intracellular ROS.

### Hcy-Induced Endothelial Dysfunction Is Mediated by COX-2

**FIGURE 5:**
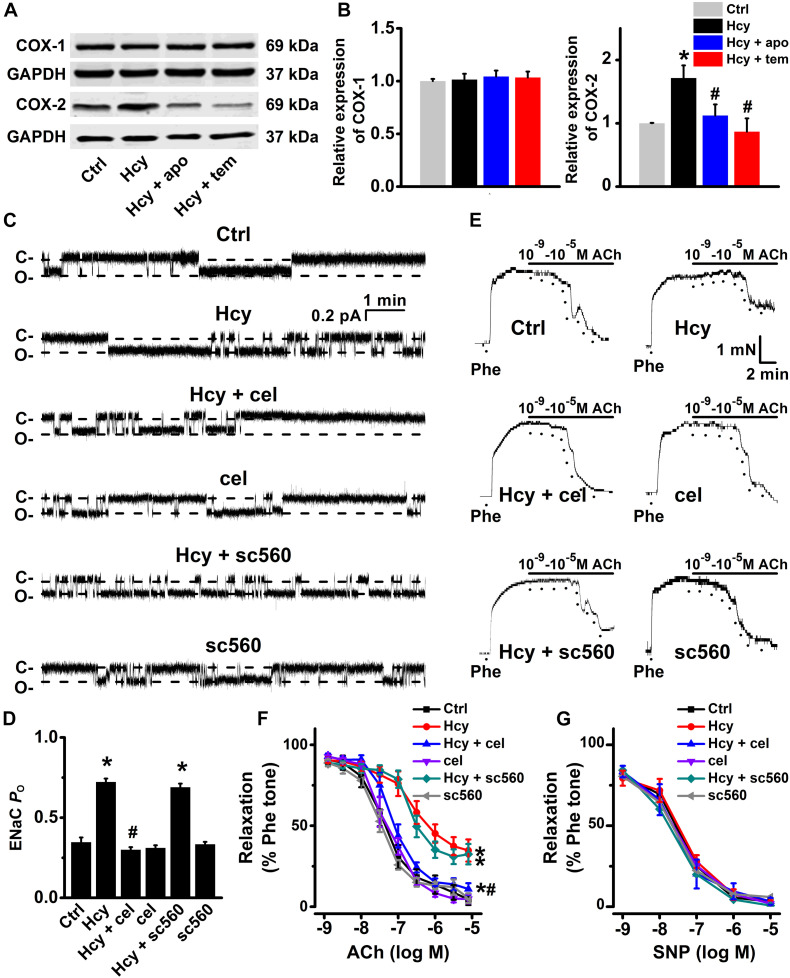
Inhibition of COX-2 by celecoxib attenuates Hcy-induced increase in ENaC activity and impairment of EDR in mouse aorta. **(A)** Representative Western blots demonstrating COX-1 and COX-2 expression in HUVECs under control condition or treated with 100 μM Hcy, 100 μM Hcy plus 100 μM apocynin (apo), or 100 μM Hcy plus 100 μM TEMPOL (tem) for 6 h, respectively. **(B)** Summary plots of Western blots, showing the expression levels of COX-1 (left) and COX-2 (right) under each indicated condition. Data are means ± SEM of six experiments in each group. *P < 0.05 vs. ctrl; #*P* < 0.05 vs. Hcy. **(C)** Representative ENaC single-channel currents recorded in endothelial cells from split-open aorta under control conditions or, respectively, treated with 100 μM Hcy, 100 μM Hcy plus 3 μM celecoxib (cel), 3 μM celecoxib alone, 100 μM Hcy plus 0.3 μM sc560, or 0.3 μM sc560 alone for 6 h. **(D)** Summarized ENaC *P*_*O*_, reflecting ENaC activity, from recordings as shown in **(C)**. Data are means ± SEM of six mice. *P < 0.05 vs. ctrl; #*P* < 0.05 vs. Hcy. **(E)** Representative traces obtained from wire myograph assays under each indicated condition and **(F)** summarized data of ACh-induced relaxation of aorta under each indicated condition. The first dot indicates the application of 10^–9^ M ACh to the 1 μM Phe precontracted arterial rings, whereas the following dots indicate ACh concentrations gradually increasing from 10^–8^.^5^ to 10^–5^ M. Data are means ± SEM of six mice. *P < 0.05 vs. ctrl; #*P* < 0.05 vs. Hcy. **(G)** Summary of artery relaxation induced by 10^–9^ to 10^–5^ M SNP in aorta from the six individual experiments under indicated conditions.

Several lines of evidence showed that Hcy significantly increased COX-2 expression in human chondrocytes (Ma et al., 2018), and human monocytes (Chien et al., 2015). Consistently, we observed that the expression levels of COX-2, but not the expression levels of COX-1, were significantly increased by exogenous 100 μM Hcy for 6 h in HUVECs and this effect was completely prevented by pre-treatment of HUVECs with either apocynin or TEMPOL (Figures 5A,B). We then next examined whether COX-2 was involved in Hcy-induced increase in ENaC activity in the intact endothelial cells of aorta. Our data showed that pre-treatment of aorta with the celecoxib, the COX-2 inhibitor, but not sc560, the COX-1 inhibitor, attenuated Hcy-induced increase in ENaC activity in these cells and that each inhibitor alone had no effect on the basal ENaC activity (Figures 5C,D). Consistently, celecoxib, but not sc560, prevented exogenous Hcy-induced impairment of mouse aortic EDR (Figures 5E,F). However, endothelial-independent relaxations in response to SNP were unaffected by exogenous Hcy, celecoxib and sc560 (Figure 5G). These data together suggest that the ROS generation and an increased COX-2 expression contribute to Hcy-induced elevation of ENaC activity, which accounts for impaired EDR in Hcy-treated aorta.

### Inhibition of TXB2 Synthesis Attenuated Hcy-Induced ENaC Activity and Impaired Endothelial Relaxation

**FIGURE 6:**
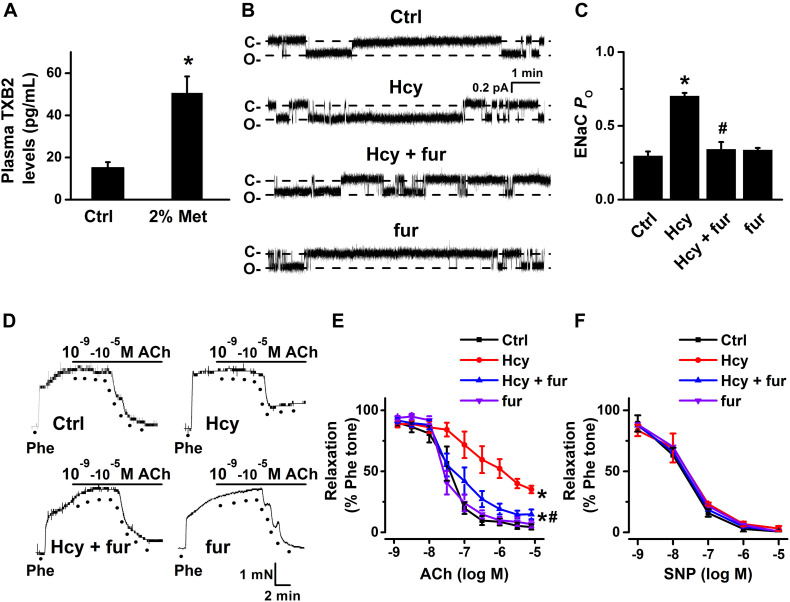
Inhibition of TXB2 by furegrelate ameliorated Hcy-induced increase in ENaC activity and impairment of EDR in mouse aorta. **(A)** Mean plasma TXB2 levels, the stable metabolic product of TXA_2_, were measured in control and L-methionine (Met)-treated mice. Data are means ± SEM of six mice. *P < 0.05 vs. ctrl. **(B)** Representative ENaC single-channel currents recorded in endothelial cells from split-open aorta either under control condition or, respectively, treated with 100 μM Hcy, 100 μM Hcy plus 10 μM furegrelate (fur), or 10 μM furegrelate alone for 6 h. **(C)** Summarized ENaC *P*_*O*_ under conditions as shown in **(B)**. Data are means ± SEM of six mice. *P < 0.05 vs. ctrl; #*P* < 0.05 vs. Hcy. **(D)** Representative traces obtained from wire myograph assays under each indicated condition and **(E)** summarized data of ACh-induced relaxation of aorta under each indicated condition. The first dot indicates the application of 10^–9^ M ACh to the 1 μM Phe precontracted arterial rings, whereas the following dots indicate ACh concentrations increasing from 10^–8^.^5^ to 10^–5^ M. Data are means ± SEM of six mice. *P < 0.05 vs. ctrl; #*P* < 0.05 vs. Hcy. **(F)** Summary of artery relaxation induced by 10^–9^ to 10^–5^ M SNP in aortas from the four individual experiments under indicated conditions.

Previous studies showed that elevation of COX-2 expression led to a dramatically increased production of TXB2 (Feletou et al., 2011). Indeed, our data showed that the mean plasma level of TXB2 was significantly increased by L-methionine in mice as compared with control (Figure 6A). Then, we further examined whether inhibition of TXB2 attenuated Hcy-induced increase in ENaC activity in the intact endothelial cells of mouse aorta. Our data showed that pre-treatment of mouse aorta with furegrelate, a thromboxane synthase inhibitor, significantly attenuated exogenous Hcy-induced activation of ENaC in aortic endothelial cells, whereas furegrelate alone had no effect on basal ENaC activity in the intact endothelial cells of mouse aorta (Figures 6B,C). Consistently, furegrelate significantly improved the Hcy-induced impairment of endothelial-dependent relaxations (aorta treated with 100 μM Hcy for 6 h), while furegrelate alone had no effect on ACh-induced relaxations in the control aorta (Figures 6D,E). In addition, endothelial-independent relaxations in response to SNP were unaffected by either Hcy or furegrelate (Figure 6F). These data suggest that COX-2-derived TXB2 mediates Hcy-induced increase in ENaC activity and impaired EDR in mouse aorta.

### Activation of SGK1/Nedd4-2 Signaling Contributes to Hcy-Induced Impairment of EDR

**FIGURE 7:**
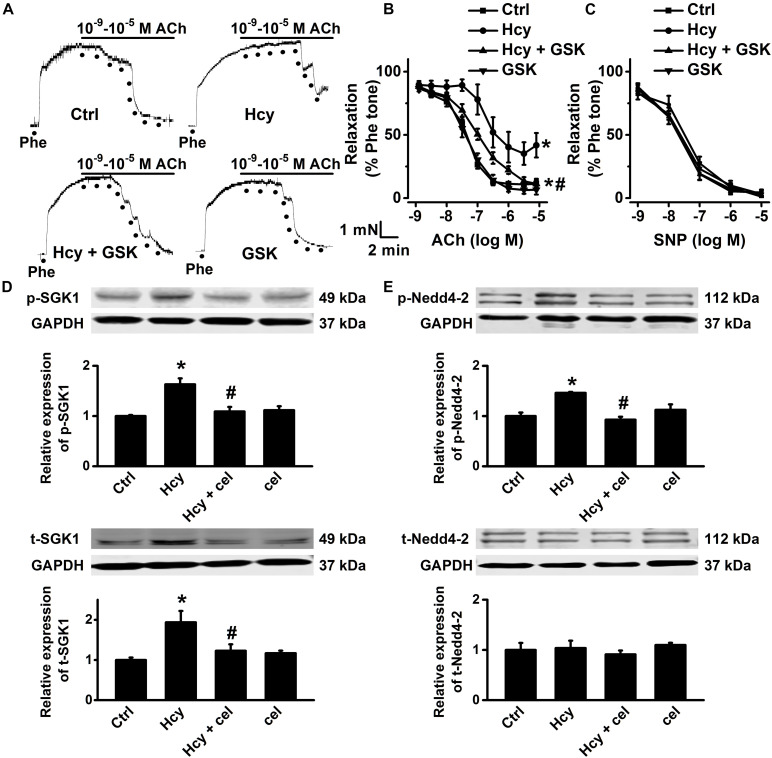
Celecoxib ameliorated Hcy-induced impairment of EDR by inhibition of SGK1/Nedd4-2 signaling. **(A)** Representative traces obtained from wire myograph assays under each indicated condition and **(B)** summarized data of ACh-induced relaxation of aorta either under control condition or, respectively, treated with 100 μM Hcy, 100 μM Hcy plus 10 μM GSK650394 (GSK), or 10 μM GSK650394 alone for 6 h. The first dot indicates the application of 10^–9^ M ACh to the 1 μM Phe precontracted arterial rings, whereas the following dots indicate ACh concentrations ranged from 10^–8^.^5^ to 10^–5^ M. Data are means ± SEM of six mice. *P < 0.05 vs. ctrl; #*P* < 0.05 vs. Hcy. **(C)** Summary of artery relaxation induced by 10^–9^ to 10^–5^ M SNP in aorta from the four individual experiments. **(D,E)** Representative Western blots demonstrating the expression of total SGK1 (t-SGK1), phosphorylated SGK1 (p-SGK1), total Nedd4-2 (t-Nedd4-2) and phosphorylated Nedd4-2 (p-Nedd4-2) under control condition or, respectively, treated with 100 μM Hcy, 100 μM Hcy plus 3 μM celecoxib, or 3 μM celecoxib alone for 6 h. Summary plots of Western blots, showing the summarized expression levels of t-SGK1, p-SGK1, t-Nedd4-2, and p-Nedd4-2 under each indicated condition. Data are means ± SEM of six experiments in each group. *P < 0.05 vs. ctrl; #*P* < 0.05 vs. Hcy.

A number of studies demonstrated that activation of SGK1/Nedd4-2 signaling stimulates ENaC activity (Kamynina and Staub, 2002; Wu et al., 2019; Yang et al., 2020). We then examined whether activation of SGK1/Nedd4-2 signaling may contribute to Hcy/COX-2 mediated impairment of EDR in mouse aorta. Our data showed that GSK650394, the SGK1 inhibitor, ameliorated Hcy-induced impairment of EDR in mouse aorta; while GSK650394 alone had no effect on EDR in control aorta (Figures 7A,B). Moreover, endothelial-independent relaxations in response to SNP were unaffected by Hcy or GSK650394 (Figure 7C). Moreover, Western blots data showed that the expression levels of phosphorylated Nedd4-2, phosphorylated SGK1, and total SGK1 expression, but not total Nedd4-2 were significantly increased by exogenous Hcy and that these effects were dramatically attenuated by celecoxib (Figures 7D,E).

**FIGURE 8:**
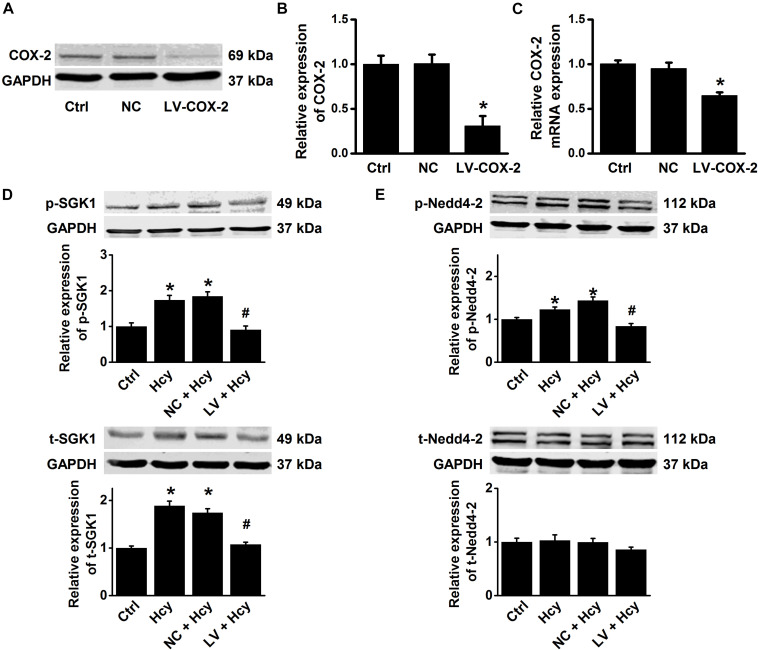
COX-2 gene silencing attenuates Hcy-induced activation of SGK1/Nedd4-2 signaling in HUVECs. **(A)** Representative Western blots demonstrating COX-2 expression in HUVECs under the conditions of control, or transiently transfected with either COX-2 control LV or COX-2 shRNA LV (MOI = 10). **(B)** Summary plots of Western blots, showing the expression levels under each indicated condition. Data are means ± SEM of six experiments in each group. *P < 0.05 vs. ctrl. **(C)** Quantification of the results obtained from Real-time PCR analysis demonstrating the levels of COX-2 mRNA expression in HUVECs under each indicated condition. Data are means ± SEM of six experiments in each group. *P < 0.05 vs. ctrl. **(D)** Representative Western blots and summarized plots demonstrating the effects of Hcy (100 μM for 6 h) on the expression levels of p-SGK1 (upper panels) and t-SGK1 (lower panels) in control HUVECs, or in HUVECs transfected with either shRNA against COX-2 or scramble shRNA. **(E)** Representative Western blots and summarized plots demonstrating the effects of Hcy (100 μM for 6 h) on the expression levels of p-Nedd4-2 (upper panels) and t-Nedd4-2 (lower panels) in control HUVECs, or in HUVECs transfected with either shRNA against COX-2 or scramble shRNA. Data are means ± SEM of six experiments in each group. *P < 0.05 vs. ctrl; ^#^*P* < 0.05 vs. Hcy.

To further confirm the role and mechanisms of COX-2 in mediating Hcy-induced ENaC activity, COX-2 gene-silencing experiments were performed in HUVECs. The data showed that the lentivirus shRNA agonist COX-2 (LV-COX-2), but not the scrambled shRNA (LV-NC), significantly reduced COX-2 expression at both the mRNA and protein levels (Figures 8A–C). We then examined the effect of COX-2 knockdown on Hcy-induced activation of SGK1/Nedd4-2 signaling in HUVECs. The results showed that the Hcy-induced increase in the expression levels of p-SGK1, t-SGK1, and p-Nedd4-2 were significantly attenuated by knockdown COX-2, but not by scramble shRNA; whereas Hcy did not affect t-Nedd4-2 expression (Figures 8D,E). These data suggest that COX-2 stimulated SGK1/Nedd4-2 signaling may account for Hcy-induced activation of ENaC, as well as impairment of EDR.

## Discussion

The major findings of the present project are as follows: HHcy led to impairment of EDR by stimulating ENaC, through promoting ROS generation and COX-2 expression mediated activation of SGK1/Nedd4-2 signaling. Our data suggest that blockade of endothelial ENaC could be a potential therapeutic strategy for HHcy-induced vascular dysfunction.

It has been reported that HHcy is an independent risk factor for cardiovascular diseases (Kalra, 2004; Mazza et al., 2004). Our previous studies showed that pathological stimuli, including high salt and oxidized LDL activated endothelial ENaC contributes to the development of vascular dysfunction and/or hypertension (Liang et al., 2018; Wang et al., 2018b; Yang et al., 2020; Niu et al., 2021). One of the mechanisms of these pathological stimuli activate endothelial ENaC is associated with excessive accumulation of ROS. More recently, we demonstrated that benzamil, a potent ENaC blocker, effectively ameliorates high fat diet-induced impairment of aortic EDR and formation of the atherosclerotic lesion, via reducing expression of proinflammatory cytokines and production of adhesion molecules in LDLr^–/–^ mice (Niu et al., 2021). These results suggest that ENaC is involved in stimulating hyperlipidemia mediated vascular inflammation. In addition, HHcy is known to regulate inflammatory responses by increasing COX-2 expression in a variety of cell types (Wu et al., 2009; Lee et al., 2013; Ma et al., 2018). Furthermore, celecoxib could greatly attenuate systemic inflammatory response in coronary artery disease (Chenevard et al., 2003). These results led us to hypothesize that HHcy leads to vascular dysfunction via activation of endothelial ENaC.

To this end, we established an experimental HHcy mouse model using dietary modification (Zhang et al., 2012), because dietary L-methionine-induced HHcy has been associated with vascular dysfunction and reduction of eNOS activity in mice (Jiang et al., 2005). Our data show that 4 weeks after L-methionine administration the levels of plasma Hcy increased significantly, which is an indication for establishing HHcy in mouse. More importantly, the aortic endothelial ENaC was greatly activated and EDR of aorta was impaired by L-methionine administration, suggesting HHcy may stimulate endothelial ENaC and subsequently induces impaired EDR in L-methionine administered mice. We then isolated and treated mouse aorta with exogenous Hcy to examine whether exogenous Hcy application could mimic the effects of L-methionine on ENaC and EDR. Consistently, exogenous application of Hcy to isolated aorta resulted in a significant increase in endothelial ENaC activity and led to an impairment of EDR, which were abolished by benzamil. These results strongly suggest that HHcy-induced activation of ENaC, in aortic endothelial cells, is tightly associated with the impairment of EDR.

Previous studies suggested that HHcy could cause endothelial injury and vascular dysfunction by accumulating intracellular ROS (Esse et al., 2019). Additionally, our recent studies showed that ROS-mediated strong activation of ENaC, in both the endothelial cells and the distal nephron principal cells, contributes to the development of hypertension (Wang et al., 2018a; Wu et al., 2019). Therefore, we reasoned that the increased intracellular ROS levels may participate in Hcy-induced increase in ENaC activity. Consistent with this notion, our data demonstrated that exogenous Hcy significantly induced accumulation of intracellular ROS in both mouse aorta and HUVECs, which were reversed by apocynin or TEMPOL, suggesting NADPH-mediated ROS accumulation plays role in Hcy-induced activation endothelial ENaC. This notion is strongly supported by the experiments, where apocynin or TEMPOL restored Hcy-induced activation of ENaC and impairment of EDR. Previous studies have shown that p-SGK1 mediated elevation of phosphorylated Nedd4-2 levels reduces the interaction between Nedd4-2 and ENaC and therefore increases the abundance of functional ENaC in the cell membrane (Debonneville et al., 2001). Moreover, we and others have shown that an elevation of ROS significantly increases SGK1 expression in both renal cortical collecting ducts principal cells and peritoneal fibroblasts (Yamahara et al., 2009; Wu et al., 2019). Therefore, we suggest that the ROS-induced increase in ENaC activity contributes to Hcy-induced vascular dysfunction.

Previous study showed that COX-2 is an inducible enzyme involved in chronic inflammation (Subedi et al., 2019) and that excessive ROS increases the expression of COX-2 in the arteries of aging, diabetic, and hypertensive rats (Shi and Vanhoutte, 2008; Shi et al., 2008; Wong et al., 2010; Tian et al., 2012). Consistently, our data showed that the expression of COX-2, rather than COX-1, was significantly increased by exogenous Hcy in HUVECs, which was reversed by apocynin or TEMPOL. These results suggest that the increased COX-2 expression may involve in Hcy-mediated activation of ENaC. This notion is supported by the data that Hcy-induced activation of endothelial ENaC and dysfunction of vascular relaxation were reversed by celecoxib, but not by COX-1 inhibitor. These findings further imply that increased intracellular ROS stimulates COX-2 expression, which is responsible for Hcy-induced activation of endothelial ENaC and vascular dysfunction.

Our recent study demonstrated that endothelial ENaC plays an important role in high-fat diet-induced atherosclerosis in LDLr^–/–^ mice, via stimulating COX-2 mediated inflammatory signaling (Niu et al., 2021). Interestingly, our data obtained from the current study further demonstrated that an increased ENaC activity is tightly linked with Hcy-induced elevation of by COX-2 expression. Furthermore, we show that Hcy-induced increase in the expression levels of p-SGK1/SGK1 and p-Nedd4-2 was dramatically inhibited by celecoxib, suggesting the involvement of COX-2 in Hcy-induced activation of ENaC through SGK1/Nedd4-2 signaling. Therefore, we argue that there is most likely a positive feedback between ENaC and COX-2 and that this positive feedback amplifies inflammatory responses under the condition of Hcy. Nevertheless, our findings also extend the pathophysiological role of COX-2 in Hcy-induced vascular dysfunction, where the endothelial ENaC plays a critical role.

TXA2 is a powerful vasoconstrictor and aggregating factor with proinflammatory properties in the cardiovascular system; its overproduction is closely associated with vascular dysfunction (de Sotomayor et al., 2005; Vessieres et al., 2010; Romacho et al., 2016). We found that the plasma levels of TXB2 were significantly increased by L-methionine in mice. This is not surprising, because it has been suggested that ROS triggers the release of TXA2 via COX-2 in high glucose-treated human aortic endothelial cells and hypertensive rat arteries (Cosentino et al., 2003; Tian et al., 2012). Our data further demonstrated that furegrelate, the thromboxane synthase inhibitor, significantly attenuated Hcy-induced ENaC activity and impairment of EDR in mouse aorta, suggesting that the release of TXA2 (as reflected by increase in its stable metabolite TXB2) plays a critical role in Hcy-induced activation of endothelial ENaC, as well as the impartment of EDR.

Recent studies suggest that high salt diet stimulates ENaC in dendritic cells, thereby leading to vascular dysfunction and hypertension (Barbaro et al., 2017; Van Beusecum et al., 2019). Therefore, we cannot completely rule out the possibility that activation of ENaC in dendritic cells may contribute to Hcy-induced vascular dysfunction. Nevertheless, our data showed that Hcy increased ROS-mediated COX-2 expression, which augments the release of TXA2; the latter elevated endothelial ENaC activity and expression via SGK1/Nedd4-2 signaling and thus impairs EDR. The present study demonstrated that inhibition of ENaC and COX-2 or ROS scavenger could be a potential therapeutic strategy to ameliorate HHcy related vascular diseases.

## Data Availability Statement

The original contributions presented in the study are included in the article/supplementary material, further inquiries can be directed to the corresponding author/s.

## Ethics Statement

The animal study was reviewed and approved by the Animal Research Ethical Committee of Harbin Medical University.

## Author Contributions

Z-RZ and M-MW were responsible for the major conception and design of the study. CL, Q-SW, XY, DZ, YS, NN, JY, B-HD, SJ, L-LT, JL, C-JY, and QS carried out the experiments. CL analyzed the data and prepared the figures. CL, M-MW, and Z-RZ drafted and revised the manuscript. All authors approved the final version of the manuscript.

## Conflict of Interest

The authors declare that the research was conducted in the absence of any commercial or financial relationships that could be construed as a potential conflict of interest.

## Abbreviations

COX-2: cyclooxygenase-2
CVDs: cardiovascular diseases
EDR: endothelium-dependent relaxation
ENaC: epithelial sodium channel
Hcy: homocysteine
HHcy: Hyperhomocysteinemia
HUVECs: human umbilical vein endothelial cells
Nedd4-2: neural precursor cell-expressed developmentally downregulated protein 4-2
*P*_*O*_: open probability
SGK1: serum/glucocorticoid regulated kinase 1
TXB2: thromboxane B2.
